# Comparative Analysis of Barophily-Related Amino Acid Content in Protein Domains of *Pyrococcus abyssi* and *Pyrococcus furiosus*

**DOI:** 10.1155/2013/680436

**Published:** 2013-09-26

**Authors:** Liudmila S. Yafremava, Massimo Di Giulio, Gustavo Caetano-Anollés

**Affiliations:** ^1^Evolutionary Bioinformatics Laboratory, Department of Crop Sciences, University of Illinois at Urbana-Champaign, Urbana, IL 61801, USA; ^2^National Center for Supercomputing Applications, Urbana, IL 61801, USA; ^3^Laboratory of Molecular Evolution, Institute of Genetics and Biophysics “Adriano Buzzati-Traverso”, CNR, 80131 Napoli, Italy

## Abstract

Amino acid substitution patterns between the nonbarophilic *Pyrococcus furiosus* and its barophilic relative *P. abyssi* confirm that hydrostatic pressure asymmetry indices reflect the extent to which amino acids are preferred by barophilic archaeal organisms. Substitution patterns in entire protein sequences, shared protein domains defined at fold superfamily level, domains in homologous sequence pairs, and domains of very ancient and very recent origin now provide further clues about the environment that led to the genetic code and diversified life. The pyrococcal proteomes are very similar and share a very early ancestor. Relative amino acid abundance analyses showed that biases in the use of amino acids are due to their shared fold superfamilies. Within these repertoires, only two of the five amino acids that are preferentially barophilic, aspartic acid and arginine, displayed this preference significantly and consistently across structure and in domains appearing in the ancestor. The more primordial asparagine, lysine and threonine displayed a consistent preference for nonbarophily across structure and in the ancestor. Since barophilic preferences are already evident in ancient domains that are at least ~3 billion year old, we conclude that barophily is a very ancient trait that unfolded concurrently with genetic idiosyncrasies in convergence towards a universal code.

## 1. Introduction

The biophysical properties of amino acids determine their use in proteins. Amino acid polarity and molecular volume are especially important for protein stability and function in hyper thermophilic and barophilic conditions. These two properties have been associated to the origins of the genetic code [1]. Thus, tracing amino acids with common physicochemical properties may help derive the conditions in which the genetic code originated. 

A method was developed previously to assign temperature and pressure asymmetry indices to amino acids [2]. These indices are based on patterns of amino acid substitution within homologous sequences of phylogenetically related organisms living in two different environmental conditions, including barophilic versus nonbarophilic and thermophilic versus nonthermophilic conditions. The temperature asymmetry index (TAI) reflects the extent to which an amino acid is preferred by hyper thermophiles and was studied in *Deinococcus radiodurans*, *Thermus thermophilus* [3], *Methanococci*, and *Bacilli* [2]. The hydrostatic pressure asymmetry index (PAI) reflects the extent to which an amino acid is preferred by barophiles; it was studied in *Pyrococcus furiosus* and *P. abyssi* [4] and recently extended to the *P. furiosus*—*P. yayanosi* and *Thermococcus kodakarensis*—*T. barophilus* pairs [5]. The strength of statistical significance of this preference allows ranking amino acids for their propensities to be used in hyper thermophilic [6] and barophilic organisms [7]. Under the hypothesis that life may have arisen in a thermobarophilic environment, such as hydrothermal vents where hot volcanic exhalations clashed with circulating hydrothermal water flows and primed early metabolic chemistries [8], we set out to use these measures of thermobarophily as tools for further exploration. 

If ancestral organisms originated in extremophilic conditions and genetics has recorded ancestral history, how did their descendants diverge over the course of evolutionary time? This divergence must be reflected in the function of proteins they use, which is nested in their three-dimensional structure [9]. The structural classification of proteins (SCOP) groups protein domains into a structural hierarchy that includes several levels of complexity: class, fold superfamily and family, from top to bottom [10]. Studies of domains, especially of fold superfamilies (FSFs), demonstrate their usefulness in investigating the divergence of organisms at the levels of molecular organization and physiological function (e.g., [11–18]). Thus, it is likely that the bias in the amino acid composition of protein sequences is reflected in protein structure, which could allow the study of the evolutionary divergence of organisms from a common thermo-barophilic ancestor. The hyper thermophilic barophile *Pyrococcus abyssi* and hyper thermophilic nonbarophile *P. furiosus* are two closely related organisms that are very suitable for such analysis due to the extreme similarity of their physiology [19] and the very early evolutionary origins of its lineages [16]. 

In this study we analyze the amino acid distributions within and outside the domain regions of protein sequences, with domains defined at FSF level of the SCOP hierarchy. We also explore the relationship between barophilic amino acid distributions in domain regions and the evolutionary age of the respective FSFs. Amino acid barophily and thermophily ranks tend to be specific to the pair of organisms for which they are documented. As mentioned above, barophily ranks have been previously described for *Pyrococcus*, but thermophily ranks have not. Thus, we focus our analysis on barophily. Results are interpreted in terms of ecological and physiological differences between the organisms to understand the process of divergence of the two species and in the context of protein history.

## 2. Materials and Methods

**Figure 1:**
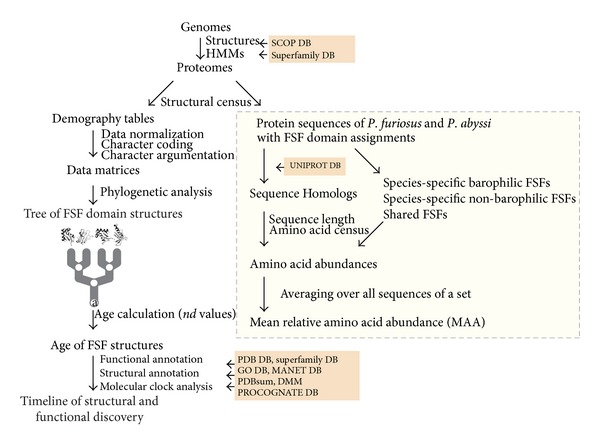
Flow chart describing the experimental strategy of the study.

FSF assignments and their respective sequences were obtained from a structural genomic census in 749 organisms [20] that used advanced linear HMMs of structural recognition in superfamily [21], probability cutoffs *e* of 10^−4^, and domain definitions from SCOP version 1.73 [10] (Figure 1). FSFs were segregated into 3 classes: (1) those present only in the barophile (species-specific barophilic FSFs), (2) those present only in the nonbarophile (species-specific nonbarophilic FSFs), and (3) those present in both species (shared FSFs). Statistical analysis of amino acid content was performed on each group, as well as on those parts of each sequence that were found outside domain assignments. A separate analysis was performed on identical FSF assignments of homologous sequences. Sequence homology was determined by mining the Uniprot database [22]. The evolutionary age of FSFs was derived directly from the phylogenomic tree of FSF domains reconstructed from the global census of protein structures (for methods see [20]). Because trees of domains are rooted and are highly unbalanced, we unfolded the relative age of protein domains directly for the phylogeny as a distance in nodes (*nd*, node distance) from the hypothetical ancestral structure at the base of the tree. *nd* was calculated by counting the number of internal nodes along a lineage from the root to a terminal node (a leaf) of the tree on a relative 0-1 scale with the following equation: *nd*_*a*_ = (number of internal nodes between nodes *r* and *a*)/(number of internal nodes between nodes *r* and *m*), where *a* is the target leaf node, *r* is the hypothetical root node, and *m* is a leaf node that has the largest possible number of internal nodes from *r*. Consequently, the *nd* value of the most ancestral taxon is 0, whereas that of the most recent one is 1. The molecular clock for protein domains at FSF level (*t* = −3.831 *nd* + 3.628) [20] was used to calculate the geological ages of selected FSFs in billions of years (Gy). Calibrations showed a significant linear correlation (*P* < 0.0001) between FSF age (*nd*) and geological time. The P-loop hydrolase fold structure, the most ancient FSF of our timeline, is used as lower boundary and linked to the earliest evidence of biological activity derived from ion microprobe analysis and isotopic composition of carbonaceous inclusions in 3.8 Gy-old banded iron rock formations. Other FSFs were linked to the biosynthesis of porphyrins (spectroscopic identification of vanadyl-porphyrin complexes in carbonaceous matter embedded in 3.49 Gy-old polycrystalline rocks), enzymes of nitrogen assimilation (with ages inferred mostly from biogeochemical evidence), lipid biomarkers such as hopanoids and biphytanes recovered from kerogen, bitumens and hydrocarbons, markers of bacterial and eukaryotic diversification episodes with times established from microfossil evidence (e.g., unicellular cyanobacterial coccoids in 1.9 Gy-old tidal sedimentary rock and acritarchs in 1.5 Gy-old rocks from Northern Australia) integrated with molecular, physiological, paleontological and geochemical data, folds linked to biological processes and lineages (e.g., biosynthesis of flavonoids and red algae, hemocyanins and mollusks), and finally present day boundary FSFs [20].

The relative abundance of each amino acid in a sequence was calculated as the number of amino acid instances divided by the total length of that sequence. The relative abundances of amino acids in domain sequences or entire protein sequences were normalized by the length of the domains or the length of entire protein sequences, respectively. These numbers were then averaged over all sequences in a group under consideration, obtaining mean relative amino acid abundance (MAA) measures specific for each amino acid. Analysis for homologous sequences was slightly different. Only the homologous sequences with identical FSF domain assignments in the two organisms were used. First, the relative amino acid abundance was calculated for each FSF assignment in each sequence. If a sequence had multiple repeats of the same FSF, the MAA values were averaged within that sequence. MAA differences were calculated for the homologous pair of sequences from the two organisms. If many sequence pairs contained a particular FSF, these MAA differences were subsequently averaged over the dataset. Statistical analyses were performed in *R* and Instat using the Welch 2-sample *t*-test. 

## 3. Results and Discussion

### 3.1. Exploring General Tendencies in Amino Acid Use

Previous work established that *P. abyssi* tends to substitute arginine (Arg), serine (Ser), valine (Val), aspartate (Asp), and glycine (Gly) for amino acids in sequences homologous to *P*.* furiosus* [4]. Henceforth we will refer to them as barophilic amino acids. Conversely, *P. furiosus* tends to substitute asparagine (Asn), lysine (Lys), proline (Pro), isoleucine (Ile), threonine (Thr), glutamine (Gln), and tyrosine (Tyr) for amino acids in sequences homologous to *P. abyssi*. Henceforth we will refer to them as nonbarophilic amino acids. The substitution of leucine (Leu), histidine (His), phenylalanine (Phe), methionine (Met), glutamate (Glu), alanine (Ala), cysteine (Cys), and tryptophan (Trp) appears to be unbiased. These preferences were determined based on the statistical significance of the underlying patterns of substitution in homologous sequences. They allow grouping amino acids into three broad categories: barophilic, nonbarophilic, and indifferent. Our task is to establish patterns of amino acid use depending on their barophilic group and location in the protein sequence. We started from the known point of reference, documenting the amino acid use in the entire protein sequences of the two organisms. From there we progressively focused on the ancestral set of functional protein sequences by studying amino acid use in domain sequences and regions that intervene between domains, then comparing amino acid counts in species-specific and shared FSF domain structures, and finally performing pairwise comparisons of amino acid use in matching domain regions of homologous sequence pairs. Finally, we compared the amino acid preferences of FSF domains that are considered most ancient and most recent from an evolutionary point of view, using ages of domain structures inferred from a structural phylogenomic census that is very well indexed [20].

### 3.2. *P. furiosus* and *P. abyssi* Are Very Similar at the Level of Protein Domain Structure and Share a Very Ancient Proteomic Ancestor

The two species of *Pyrococci* share the majority (452) of their FSF domain structures (Table 1). *P. abyssi* has fewer FSFs (472) than *P. furiosus* (495), probably because it inhabits an extremophilic niche that combines extreme pressure and temperature, both of which have been shown to put limits on viable protein structures (e.g., [23, 24]). The similarities in FSF content of proteomes are explained by the common biological heritage and similar physiology of the two organisms, which differ mainly in the utilization of metabolic substrates [19]. These differences must be reflected in the function of FSFs that are specific to each of the two organisms. To evaluate this functional link, we annotated molecular functions using FSF assignment definitions of the superfamily database [15]. Out of 20 FSFs specific to *P. abyssi*, 8 participate in “metabolism”, 6 in “regulation”, 2 in “intracellular processes”, and 4 belong to the “general” and “other” categories. Out of 43 FSFs specific to *P. furiosus*, 21 participate in “metabolism”, 6 in “regulation”, 5 in “intracellular processes” and 1 in “extracellular processes”, and 6 belong to the “general”, “other,” and “unknown” categories. As expected, the majority (30–40%) of the species-specific FSFs participate in metabolic functions. 

**Figure 2:**
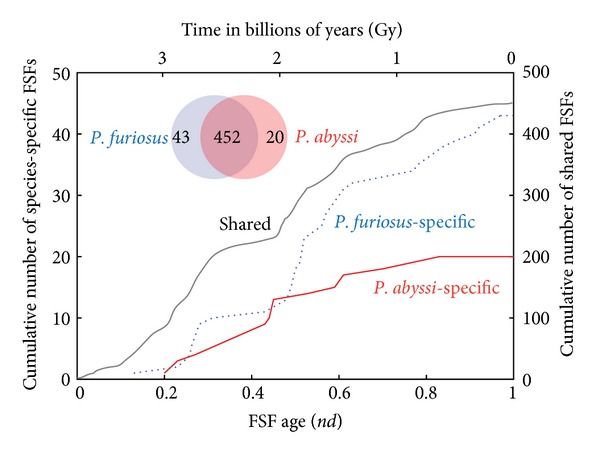
Evolutionary accumulation of protein domain structures defined at FSF level of the structural hierarchy in SCOP. The cumulative plot divides FSFs into those that are shared between *Pyrococcus* and those that are species-specific. Domain age is provided in a relative scale (*nd*, node distance) or in timescales in billions of years (Gy) according to a molecular clock of FSFs. The Venn diagram describes the actual FSF counts in the proteomes of *P. furiosus* and *P. abyssi*.

The history of divergence of protein structure and function has been previously studied with cumulative plots of protein domain structures that display the rate at which organisms accumulate domains over the course of evolutionary history [16, 25, 26]. This method is useful for the study of the diversification of organisms (Figure 2). The cumulative plot of species-specific FSFs for the two *Pyrococci* indicates early divergence both in structure and in function. This result is congruent with other studies that suggest early divergence based on sequence phylogeny and gene loss [19] and a very ancestral trend of loss in protein domain repertoires of Archaea responsible for their very early origin [16]. Since the age of FSF domain structures follows a tight molecular clock [20], it was possible to establish a timeframe for the appearance of lineage-specific FSFs in evolution. *P. furiosus*-specific FSFs appeared for the first time ~3.2 Gy ago and were closely followed by *P. abyssi*-specific FSFs, which appeared ~3 Gy ago. The timeline therefore suggests an early hyper thermophilic origin of the barophilic organism (by domain loss [16]), regardless of the accuracy of geological time assignments. However, lineage-specific FSFs are indicative of the origin of the organism and not the origin of barophilic traits. We also note that the acquisition of lineage-specific FSFs by *P. abyssi* is slower than *P. furiosus*, probably due to greater constraints imposed on *P. abyssi* by its barophilic environment. A fully enzymatic biosynthetic pathway for purine biosynthesis and a functional ribosome were already in place ~3 Gy ago during the rise of the *P. abyssi* lineage, fulfilling the expanding matter-energy and processing needs of genomic information [26]. During that time, aminoacyl-tRNA synthetases accreted anticodon-binding domains [27], unfolding the specificity of the genetic code and biasing amino acid composition of flexible regions in protein structure [28]. We note that Archaea suffered very early and impactful evolutionary episodes of genomic reduction [16, 17], setting archaeal organisms apart from those in other superkingdoms. These episodes and their associated “loser trends” most likely manifested differently but consistently in the archaeal lineages that were compared, without artificially pushing the age of lineage-specific domain repertoires significantly back in time [16].

### 3.3. The Two *Pyrococci* Display Expected Bias in Their Use of Amino Acids

**Figure 3:**
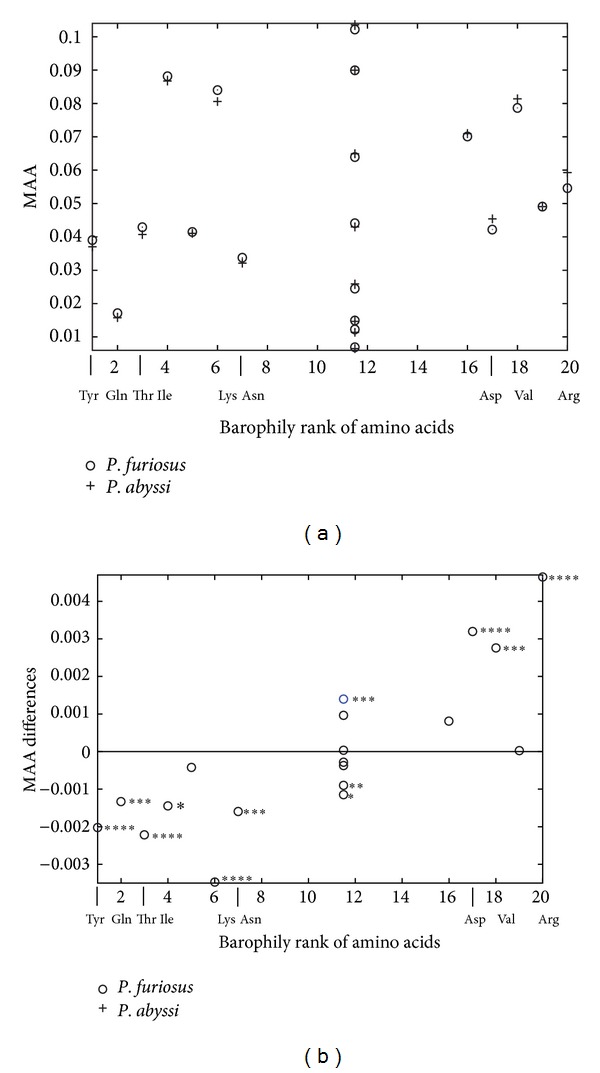
Mean relative amino acid abundance (MAA) analysis of the set of entire polypeptide sequences. (a) On average, both *Pyrococci* seem to use barophilic and nonbarophilic amino acids to the same extent. (b) The barophilic Asp, Val, and Arg are used significantly more in sequences of the barophilic *P. abyssi* compared to the nonbarophilic *P. furiosus* (positive difference), whereas the nonbarophilic Tyr, Gln, Thr, Ile, Lys, and Asn are used significantly more in *P. furiosus* compared to *P. abyssi* (negative difference). Statistical significance is marked with stars according to Welch's two-sided test: *P < 0.1; **P < 0.01; ***P < 0.001; ****P < 0.0001. A total of 1,896 *P. abyssi* and 2,125 *P. furiosus* sequences were analyzed.

The mean relative amino acid abundances (MAA) were plotted against barophily rank (BR) for the sequence of entire polypeptide chains. There does not seem to be a particular preference within *P. abyssi* for using more barophilic amino acids than nonbarophilic ones or within *P. furiosus* for using more nonbarophilic amino acids than barophilic ones (Figure 3(a)). However, BR was determined as a measure of comparison between the two species, not within them. Thus, we plotted mean MAA differences between the two *Pyrococci* by subtracting MAA of the nonbarophile from those of the barophile. A positive value thus indicates a bias for using an amino acid in *P. abyssi* relative to *P. furiosus*; a negative value indicates the bias for the reverse.  Figure 3(b) demonstrates a clear and statistically significant bias toward barophilic amino acids in *P. abyssi* and a significant bias toward nonbarophilic amino acids in *P. furiosus*. No such bias exists for the “indifferent” amino acids. This is an expected result congruent with the definition of barophily rank. Further exploration focuses on functionally important portions of the protein sequences, progressively moving the analysis closer to the evolutionary ancestor of the two species.

### 3.4. Bias in the Use of Amino Acids between the Two *Pyrococci* Is due to Their Shared FSF Repertoires

The apparent functional similarities pose a question: does the above bias of amino acid abundance within complete protein sequences arise from the parts of those sequences that correspond to protein domains or the intervening “connecting” sequences between domains? It is logical to predict that the latter have smaller, if any, bias, compared to the FSF domain regions. Indeed, the surmised purpose of the amino acid substitutions in barophiles is to stabilize domain structure against penetration by water, which tends to be forced into the protein core under the high pressure of the ocean abyss [23]. The effect on the intervening and more flexible regions should therefore be negligible.

**Figure 4:**
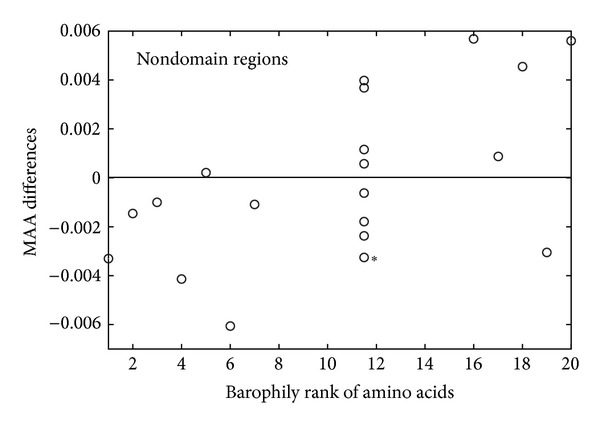
MAA analysis of nondomain regions shows there is no significant difference in barophilic and nonbarophilic amino acid use between the two organisms. Statistical significance is marked with stars as described in Figure 2. A total of 1,229 *P. abyssi* and 1,339 *P. furiosus* sequences were analyzed.

**Figure 5:**
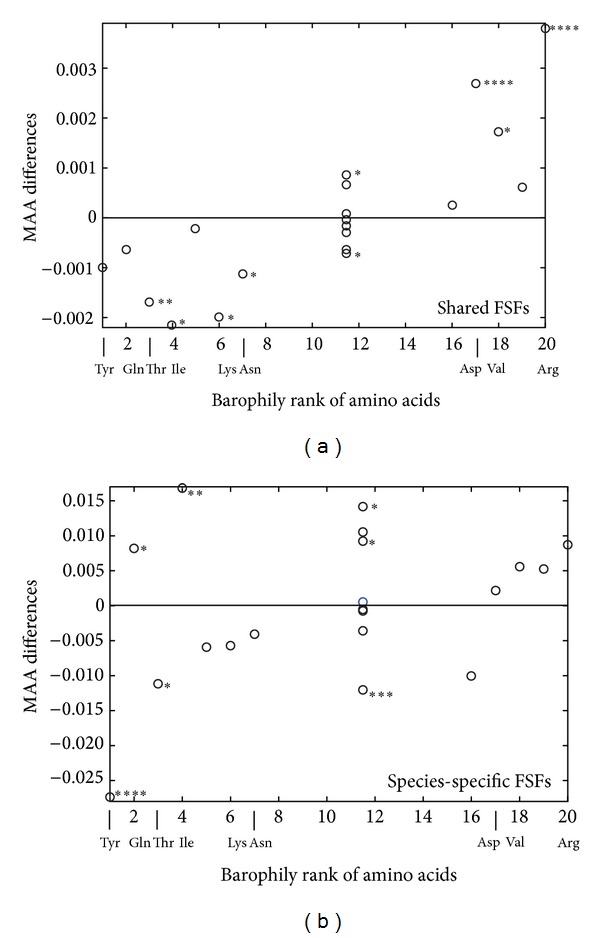
MAA analysis of shared FSF domains. (a) Although Tyr and Gln were used significantly more by *P. furiosus* in entire sequences, their use within the shared FSF domains seems to be statistically the same between the two *Pyrococci*. (b) Amino acids do not follow their patterns of homologous substitution within the species-specific FSFs. Statistical significance is marked with stars as described in Figure 2. A total of 1,627 *P. abyssi* and 1,739 *P. furiosus* sequences were analyzed.

As expected, the statistics on MAA differences computed within the intervening sequences showed no significant difference in amino acid use between the two organisms (Figure 4). In contrast, regions of FSFs shared by the two organisms show significant bias toward using barophilic amino acids in *P. abyssi* and nonbarophilic amino acids in *P. furiosus* (Figure 5(a)). This stands for all amino acids displaying statistical significance in whole sequences, except Gln and Tyr. It is interesting to note that this bias is broken within the species-specific FSF regions (Figure 5(b)), where nonbarophilic Ile and Gln change alliances, being present in higher abundance in the barophilic *Pyrococcus*. These patterns may be explained by two non-competing hypotheses. (1) The specific FSF domains likely arose in these organisms after the divergence from the common ancestor, and consequently, their evolution proceeded de novo according to the idiosyncratic ecological conditions of each species. Thus, their use of amino acids is not subject to the rules of homologous amino acid substitution, and the BR measure is inapplicable to their case. (2) The shared FSFs are more likely to belong to homologous sequences that both organisms inherited from their common ancestor. Their evolution proceeded from a certain starting point, which may have not been optimal for functioning in their eventual ecological niche. Amino acid substitutions were therefore used to stabilize the function of respective proteins, resulting in the observed patterns. These hypotheses naturally lead us to the exploration of the homologous sequences and their FSF domains. 

### 3.5. Analysis of Homologous Sequence Pairs Sharing FSF Domains

We found a total of 359 pairs of homologous sequences in the two *Pyrococci*. These sequences fall into 5 different categories:

1. 29 pairs in which neither sequence has FSF assignments,
2. 9 pairs in which FSF assignments were made in one sequence but not in the other,
3. 10 pairs in which extra FSF assignments were made in one of the sequences of a pair,
4. 3 pairs with completely different FSF assignments,
5. 309 pairs with identical assignments.

**Figure 6:**
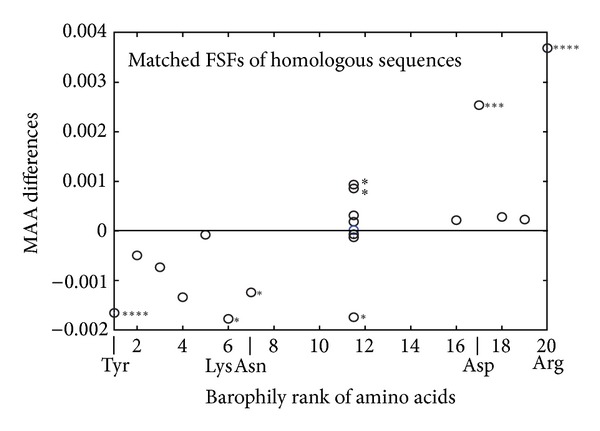
MAA analysis of matching FSF domains within pairs of homologous sequences confirms significant preferences of the barophilic organism for Arg and Asp and a preference of the nonbarophilic organism for Tyr, Lys, and Asn. Other barophilic and nonbarophilic amino acids do not display a significant difference in use. Statistical significance is marked with stars as described in Figure 2. A total of 241 matching FSFs were analyzed.

Categories (1) and (2) are not useful for comparing amino acid content of FSF domains. None of the species-specific FSFs were found in categories (3) and (4), supporting our first hypothesis that specific FSF were developed de novo. Thus, we proceeded to investigate MAA differences within the matching FSFs of the homologous sequences from category (5). Results demonstrate similar biases in use of amino acids as were found in the preceding comparisons (Figure 6). However, Val, Ile, Thr, and Gln lost the statistical significance they had in the total set of FSFs. This may have happened for at least two reasons: either these amino acids are the most representative of the ancestral sequences and the most difficult to change or they are important for novel functions introduced at later time during the process of divergence. 

**Figure 7:**
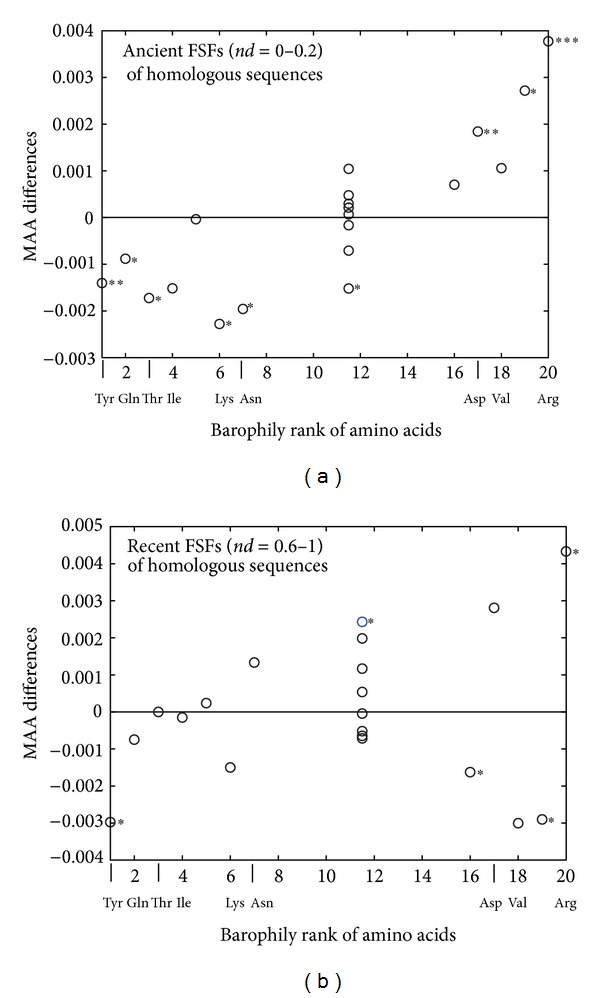
MAA analysis of matching FSFs of different evolutionary age. (a) Organisms display the same preferences for amino acids within ancient FSFs (*nd* = 0–0.2) of homologous sequences as they do in entire sequences. However, here Ser is also preferred more by the barophile. (b) Many amino acid preferences are erased in evolutionarily recent FSFs (*nd* = 0.6–1.0) of homologous sequences. Some preferences were even reversed; the normally barophilic Gly and Ser are now used significantly more by the nonbarophilic organism. Statistical significance is marked with stars as described in Figure 2. A total of 55 recent and ancient FSFs were analyzed.

To tease these two possibilities apart, we identified a set of the most ancient FSFs and a set of the most recent ones. The evolutionary age of FSF domains has been established previously in the works of Wang et al. [16, 20, 25] by building a phylogenetic tree of FSFs based on their global abundance in organisms. The evolutionary age of each FSF was calculated by tracing the number of internal nodes (*nd*) for each FSF lineage, starting from the root of the phylogenomic tree and expressed on a relative scale from 0 (the origin of protein domains) to 1 (the present). In Wang et al. [16] the most ancient FSFs have been defined as those that emerged before the beginning of massive FSF loss in the three superkingdoms due to reductive evolution (*nd* = 0–0.2). The choice of ancestry values for ancient FSFs in *Pyrococcus* is also supported by the history of their divergence: at *nd* = 0.2 both species have acquired unique FSFs (Figure 2). For the group of more recent FSFs we chose those that emerged after the “big bang” of domain combination [25], which is mostly driven by Eukarya (*nd* = 0.6–1). Comparison of MAA differences between these two groups was instructive (Figure 7). It demonstrated that some amino acids have different patterns of use in FSFs of different age. While nonbarophilic Gln, Thr, Lys, and Asn were used more by *P*. *furiosus* in the ancient FSFs, their use became more balanced in the new FSFs. Similarly, the barophilic Asp, and Arg were used more by *P. abyssi* in the old FSFs, but they became more balanced in the recent FSFs. Interestingly, bias reversed completely for the barophilic Ser, which only showed significant difference in use in ancient FSFs of homologous sequence pairs. Thus, the loss of statistical significance of barophilic Val and nonbarophilic Thr, Gln, and Ile of Figure 6 can be explained by their likely relevance to unfold newly introduced functions late in evolution, even when portraying an ancestral composition that is refractory to change (e.g., Thr and Gln).

We note that the impact of horizontal gene transfer (HGT) or other convergent evolutionary processes should be considered minimal at the FSF structural level and should not affect the conclusions of this study. The structures of protein domains are retained over long evolutionary time frames and their gain or loss have been shown to be rare in lineages at FSF and other levels of the structural abstraction (e.g., [29, 30]). Structural cores evolve linearly with amino acid substitutions per site and at 3–10 times slower rates than sequences [31]. This high conservation highlights the slow dynamics of structural change in proteins and the clock-like behavior of FSF evolution [20]. The effects that lateral transfer could have on the highly conserved sequences of shared repertoires must therefore be considered negligible. Furthermore, the divergence time between the archaeal lineages examined in this study is too short compared to divergences occurring across the entire evolutionary spectrum. This fact diminishes any possible effects that HGT could have on relative amino acid abundances of lineage-specific sequences.

## 4. Conclusions

The patterns in amino acid use presented here suggest further depth to our understanding of the barophilic rank of amino acids. Previous studies designated five amino acids to be preferentially barophilic, based on the significance of their substitution patterns: Arg, Ser, Val, Asp and Gly. In our analysis only two out of these amino acids display this preference consistently and significantly in entire sequences, shared FSFs, matched FSFs of homologous sequence pairs, and ancient FSFs: Asp and Arg (Table 2). Since barophilic preferences are already evident in the ancient set of FSFs that are at least ~3 Gy-old, but not in species-specific and young domains, we conclude that barophily is a very ancient trait that goes back to the start of organismal diversification. These two amino acids appear late in the evolution of the amino acid charging function of the genetic code as judged by the age of isoacceptor tRNA [32] and coevolving synthetase domains with amino acid editing functions [28]. However, a consensus chronology of amino acid evolution placed Asp and Arg fourth and tenth in the timeline [33], suggesting that the coding of Asp could have unfolded early. In fact, Asp belongs to an early group of amino acid codified by codons of the GNN type; an observation that supports the coevolutionary theory of the origin of the genetic code [34]. While the apparent mismatch of data can be explained by separate histories of amino acid charging and encoding [32], it is clear that the primordial barophilic trait has impacted the early evolution of genetics. Since tRNA, the genetic code and Archaea appear to have polyphyletic origins (e.g., [30, 35]); results suggest the colonization of barophilic environments by the ancestors of the emerging archaeal lineages as these were unfolding genetic idiosyncrasies in convergence towards a universal code. 

Seven amino acids, Asn, Lys, Pro, Ile, Thr, Gln, and Tyr, have been previously designated as nonbarophilic. Asn, Lys, and Thr displayed a preference for non-barophily most consistently, in entire sequences, shared FSFs, matched FSFs of homologous sequence pairs, and ancient FSFs. It is possible that these “faithful” amino acids are either easy to swap within the homologous sequences as they diverge or contribute the most to the stability and function of proteins at their respective environmental conditions. Their presence in the ancient FSFs confirms the ancestrality of nonbarophilic traits, which does not invalidate the primordial nature of barophily.

An archaic nonbarophilic trait challenges the views of an abiotic start of genetics in deep vent environments occurring prior to organismal diversification [36], supporting instead the view that ocean abysses played an important role in tailoring the diversification of genetics and life [4, 5]. Consequently, the origin of primordial life (prior to the genetic code) is more likely in nonbarophilic environments such as terrestrial anoxic geothermal fields [37] or alkaline aquifers generated by serpentinizing rocks [38]. We stress that this does not nullify the fact of a crucial involvement of barophily during the rise of cellular organisms, modern biochemistry, and genetics.

The other amino acids displayed more balanced use in the sequences we inspected. This may indicate that they are most representative of the composition of the ancestral organism and are most difficult to change for structural or functional reasons. The “changeling” amino acids Ser, Ile, and Gln were preferentially used according to their BR in shared/ancient FSFs but had opposite patterns of use in specific/recent FSFs. This may indicate that their BR values have more to do with the legacy left over from the common pyrococcal ancestor, yet they are actually more useful under conditions opposite than those suggested by BR. Finally, the nonbarophilic Pro demonstrated no significant difference in any of our tests. Pro is enriched in structured regions that involve turns, which have been suggested important for primordial coding [39] and linked to loops and protein flexibility [28]. Their role in protein structure and genetics may be archaic and hardwired into the make up of proteins.

## Figures and Tables

**Table 1:** Protein sequences and FSF domain statistics.

Statistics	*P. furiosus* DSM 363	*P. abyssi* GE5
Number of sequences with FSF assignment	1,339 (63%)	1,229 (65%)
Average sequence length in base pairs ± SD (minimum–maximum length)	276 ± 181 (21–1,740)	289 ± 190 (18–2,122)
Fraction of amino acids found in FSFs (not in intervening sequences)	61%	61%
Total FSFs	495	472
Species-specific FSFs	43	20
Average number of amino acids in FSFs (minimum–maximum length)	267 (5–1,107)	272 (5–1,107)

**Table 2:** Barophily rank (BR) and preference of the barophilic (B) and non-barophilic amino acids (N). Bold font identifies amino acids that have most consistent and significant preference that is congruent with their barophily or non-barophily group.

Amino acid	BR	Complete sequence	Shared FSFs	Specific FSFs	Homologous sequences		
					All FSFs	Ancient FSFs	Recent FSFs
**Arg (B)**	**20**	**B**	**B**		**B**	**B**	
Ser (B)	19					B	N
Val (B)	18	B	B				
**Asp (B)**	**17**	**B**	**B**		**B**	**B**	
Gly (B)	16						N
**Asn (N) **	**7**	**N**	**N**		**N**	**N**	
**Lys (N)**	**6**	**N**	**N**		**N**	**N**	
Pro (N)	5						
Ile (N)	4	N	N	B			
**Thr (N)**	**3**	**N**	**N**	**N**		**N**	
Gln (N)	2	N		B		N	
**Tyr (N)**	**1**	**N**		**N**	**N**	**N**	**N**
